# Comments on: Huang et al. (2019) Emerging trends and research foci in gastrointestinal microbiome’, *J. Transl. Med.*, 17: 67

**DOI:** 10.1186/s12967-020-02379-9

**Published:** 2020-06-25

**Authors:** Yuh-Shan Ho

**Affiliations:** Trend Research Centre, Asia University, No. 500, Lioufeng Road, Wufeng, Taichung, 41354 Taiwan

**Keywords:** SCI-EXPANDED, Searching keywords, Bibliometric, Web of science category

Huang et al. recently published a paper in *Journal of Translational Medicine* entitled “Emerging trends and research foci in gastrointestinal microbiome” [1]. Huang et al. mentioned in section Methods, that ‘We obtained a record of 2891 manuscripts published between 1998 and 2018 from the Web of Science Core Collection (WoSCC) of Thomson Reuters; this record was obtained on June 23, 2018. The WoSCC is the most frequently used source of scientific information. We used the term “Gastrointestinal Microbiomes” and all of its hyponyms to retrieve the record, and restricted the subjects to gastroenterology and hepatology.’

There is no “Web of Science Core Collection (WoSCC) of Thomson Reuters” but Web of Science Core Collection of Clarivate Analytics (formerly known as the Thomson Reuters and the Institute for Scientific Information). The data were collected on June 23 in 2018. A bias might be obtained as some publications in 2018 that have not yet been updated in the Web of Science Core Collection. Using the same method as mentioned in the original paper with searching keywords “gastrointestinal microbiomes” in Topic [1] resulted only one meeting abstract entitled “Microbial disease signatures characterize the gastrointestinal microbiomes in irritable bowel syndrome” [2] in Web of Science category of gastroenterology and hepatology. When we used searching strategy by search keywords gastrointestinal microbiomes or gastrointestinal microbiome that means gastrointestinal and microbiomes or gastrointestinal and microbiome in Topic field, results 2188 documents including 339 in the category of gastroenterology and hepatology. These results show a huge difference from the results in the original paper [1].

Web of Science Core Collection includes.

Web of Science Core Collection: Citation Indexes includes.

- 1. Science Citation Index Expanded (SCI-EXPANDED).
- 2. Social Sciences Citation Index (SSCI).
- 3. Arts & Humanities Citation Index (A&HCI).
- 4. Conference Proceedings Citation Index—Science (CPCI-S).
- 5. Conference Proceedings Citation Index—Social Science & Humanities (CPCI-SSH).
- 6. Book Citation Index—Science (BKCI-S).
- 7. Book Citation Index—Social Sciences & Humanities (BKCI-SSH).
- 8. Emerging Sources Citation Index (ESCI).

Web of Science Core Collection: Chemical Indexes.

- 1. Current Chemical Reactions (CCR-EXPANDED).
- 2. Index Chemicus (IC).

The subject of gastroenterology and hepatology can only be found in SCI-EXPANDED. In addition, since there are many levels of databases as mentioned above, the authors should choose the appropriate databases for their research [3]. For instance, Emerging Sources Citation Index (ESCI) complements the highly selective indexes by providing earlier visibility for sources under evaluation as part of SCIE, SSCI, and A&HCI’s rigorous journal selection process (https://liu.brooklyn.libguides.com/az.php?a=e) [4]. SSCI, A&HCI, ESCI, CPCI-S, CPCI-SSH, BKCI-S, BKCI-SSH, CCR-EXPANDED, and IC are inappropriate for “Emerging trends and research foci in gastrointestinal microbiome” [1].

From my view, Huang et al. used inappropriate searching keywords and method to publish bibliometric paper in *Journal of Translational Medicine*, this may result in misleading the journal readers. The authors could have provided a greater accuracy and information about their data if they understood Web of Science Core Collection beforehand. In addition, the fact that only 339 documents were published in 21 years (1998–2018) is not legitimate for a statistics point of view.

## Data Availability

Not applicable.

## Abbreviations

SCI-EXPANDED: Science Citation Index Expanded
SSCI: Social Sciences Citation Index
A&HCI: Arts & Humanities Citation Index
CPCI-S: Conference Proceedings Citation Index—Science
CPCI-SSH: Conference Proceedings Citation Index—Social Science & Humanities
BKCI-S: Book Citation Index—Science
BKCI-SSH: Book Citation Index—Social Sciences & Humanities
ESCI: Emerging Sources Citation Index
CCR-EXPANDED: Current Chemical Reactions
IC: Index Chemicus

## References

[1] Huang XQ, Fan XWW, Ying J, Chen SY (2019). Emerging trends and research foci in gastrointestinal microbiome. J Transl Med.

[2] Versalovic J, Saulnier DM, Richle K, Mistretta TA, Diaz MA, Raza S, Weidler EM, Milosavljevic A, Coarfa C, Mandal D, Lynch S, Petrosino J, Highlander S, Gibbs R, Shulman R (2011). Microbial disease signatures characterize the gastrointestinal microbiomes in irritable bowel syndrome. Gastroenterology.

[3] Ho YS (2019). Comments on the Reply to the Rebuttal to: Zhu, Jin, & He ‘On evolutionary economic geography: a literature review using bibliometric analysis’. European Planning Studies vol. 27, pp 639–660. Eur Plan Stud.

[4] Ho YS (2019). Comment on Chen, J., Su, Y., Si, H., Chen, J. Managerial areas of construction and demolition waste: A scientometric review. Int. J. Environ. Res. Public Health 2018, 15 (11), 2350. Int J Environ Res Public Health..

